# Effectiveness and Safety of SARS-CoV-2 Vaccines among Children and Adolescents: A Systematic Review and Meta-Analysis

**DOI:** 10.3390/vaccines10030421

**Published:** 2022-03-10

**Authors:** Peng Gao, Shan Cai, Qiao Liu, Min Du, Jue Liu, Min Liu

**Affiliations:** 1 Department of Epidemiology and Biostatistics, School of Public Health, Peking University, Beijing 100191, China; 1710306136@pku.edu.cn (P.G.); 1610306236@pku.edu.cn (Q.L.); 1510306111@pku.edu.cn (M.D.); jueliu@bjmu.edu.cn (J.L.); 2 Institute of Child and Adolescent Health, School of Public Health, Peking University, Beijing 100191, China; 1710306207@pku.edu.cn; 3 Institute for Global Health and Development, Peking University, Beijing 100871, China

**Keywords:** SARS-CoV-2, vaccine, safety, effectiveness, children, adolescents

## Abstract

Background: The proportion of children and adolescents with COVID-19 had gradually increased according to data reported by WHO. However, there was no meta-analysis of effectiveness and safety of SARS-CoV-2 vaccines in children and adolescents. We aimed to provide investigation-based medical evidence and reference recommendations for children and adolescents in regard to SARS-CoV-2 vaccines. Methods: We systematically searched PubMed, Embase, and Web of Science from inception to 5 January 2022. RCTs and observational studies that examined the effectiveness and safety were included. Results: A total of 13 eligible studies were included for analysis. For the first dose, the effectiveness of SARS-CoV-2 vaccines against SARS-CoV-2 infection and COVID-19 was 88.5% (95% CI:15.7–98.4%, *p =* 0.033) and 84.3% (95% CI: 66.6–92.6%, *p <* 0.001) separately. For the second dose, the effectiveness against SARS-CoV-2 infection and COVID-19 was 91.6% (95% CI: 37.8–99.5%, *p =* 0.083) and 92.7 (95% CI: 82.2–97.0, *p <* 0.001) separately. Injection-site pain, fatigue, headache, anorexia, and axillary swelling were the top five adverse events after the first dose of SARS-CoV-2 vaccines. Fatigue, injection-site pain, headache, chills, and myalgia/muscle pain were the top five adverse events after the second dose of SARS-CoV-2 vaccines. Conclusions: SARS-CoV-2 vaccines had good effectiveness and safety in children and adolescents. We suggest that children and adolescents should get vaccinated as soon as possible to protect themselves and slow the spread of the pandemic.

## 1. Introduction

Severe Acute Respiratory Syndrome Coronavirus 2 (SARS-CoV-2) is a new strain of coronavirus that emerged in 2019, and it has caused a pandemic of Corona Virus Disease 2019 (COVID-19) in the world. The current global epidemic situation is still severe and has not yet been effectively controlled. According to the latest report of the World Health Organization (WHO), as of 14 January 2022, the number of new cases worldwide in the past 24 h was 3,120,435, with a total of almost 319 million cumulative confirmed cases, and a total of over 5 million cumulative deaths [1]. For COVID-19, symptomatic and supportive treatments are widely used at present, and the WHO suggests some traditional drugs, such as hydroxychloroquine, lopinavir/ritonavir, and corticosteroids, as treatments, while some hospitalized patients can also use specific drugs, such as remdesivir [2]. SARS-CoV-2 vaccines vaccination is an effective way to prevent SARS-CoV-2 infections. Dozens of SARS-CoV-2 vaccines have been approved for use around the world. The WHO has determined that the following vaccines against COVID-19 have met the necessary criteria for safety and effectiveness: BNT162b2, mRNA-1273, AZD1222, Covishield, Ad26.COV2.S, BBIBP-CorV, and CoronaVac vaccines [3]. Moreover, According to statistics, as of 13 January 2022, nearly 4.6 billion persons in the world had received at least one dose of the vaccines, and 3.9 billion persons had completed full vaccination [4].

Compared with adults, the clinical manifestations of children and adolescents infected with SARS-CoV-2 are usually mild or asymptomatic, but there are still a small number of severe infections, leading to hospitalization and even death. According to the American Academy of Pediatrics, as of 15 January 2022, the United States had reported a total of over 65 million cumulative confirmed cases and a total of 847,577 cumulative deaths among children and adolescents [5]. As SARS-CoV-2 vaccines are developed and gradually widely used worldwide, the increase in cases of unvaccinated children and adolescents is worthy of attention. According to WHO monitoring data, the proportion of children and adolescents with COVID-19 had gradually increased, and the proportion of cases aged <5 and 5 ~ 14 increased from 1% and 2.5% in January 2020 to 2% and 8.7% in July 2021, respectively [4].

At present, in order to effectively control the spread of SARS-CoV-2 in the population, Canada, the United States, China, Europe, Singapore, the United Arab Emirates, Kuwait, and other countries have successively approved the emergency vaccination of SARS-CoV-2 vaccines in children and adolescents [6]. The effectiveness and safety of SARS-CoV-2 vaccines in children and adolescents are the focus of attention. So far, a number of randomized clinical trials (RCTs) [7,8,9,10,11,12] and observational studies [13,14,15,16] have been carried out worldwide, but there is only a qualitative systematic review [17], making a descriptive analysis of the relevant published and ongoing clinical studies without statistical analysis. Therefore, the purpose of this article is to collect and analyze the published studies and systematically evaluate the effectiveness and safety of SARS-CoV-2 vaccines in children and adolescents in order to provide investigation-based medical evidence and reference recommendations for children and adolescents in regard to SARS-CoV-2 vaccines.

## 2. Materials and Methods

### 2.1. Search Strategy

Our study was conducted in strict accordance with the Preferred Reporting Items for Systematic Reviews and Meta-Analysis (PRISMA) and Meta-Analyses of Observational Studies in Epidemiology (MOOSE) guidelines. The study protocol has been registered with the Prospective Register of Systematic Reviews (PROSPERO, CRD42021289931).

We systematically searched the PubMed, Embase, and Web of Science databases to collect the literature on SARS-CoV-2 vaccines among children and adolescents, from their inception to 5 January 2022. We used the following combinations as search terms: (COVID-19 OR SARS-CoV-2 OR coronavirus) AND (vaccination OR vaccine) AND (children OR childhood OR Infant OR adolescent OR adolescence OR teenager OR youth) AND (effectiveness OR safety) (see detailed search strategy in Supplementary Table S1).

### 2.2. Inclusion and Exclusion Criteria

We used EndNoteX9.3 (Tomson ResearchSoft, Stanford, CA, USA) to manage records, exclude duplicates, and screen the literature strictly according to the following inclusion and exclusion criteria: we basically included studies that examined the effectiveness and safety of SARS-CoV-2 vaccines among children and adolescents, including RCTs and observational studies. The following types of studies were excluded: (1) reviews, books, editorials, conference papers, clinical guidelines, comments, animal experiments, or case reports; (2) those that were irrelevant to the subject of the systematic review and meta-analysis, such as studies that did not use SARS-CoV-2 vaccines among children and adolescents; (3) those with insufficient data to calculate the rate and outcomes for the effectiveness and safety of SARS-CoV-2 vaccines among children and adolescents; (4) duplicate studies or overlapping participants; and (5) studies that did not clarify the identification of COVID-19. For example, the confirmed diagnosis of COVID-19 via reverse-transcription polymerase chain reaction (RT-PCR) test, serologic test, or other means was not mentioned in the text.

The literature screening was divided into three processes: Firstly, we used EndNoteX9.3 (Clarivate, London, UK) to remove duplicate works from the literature. Then two researchers independently screened the literature by reading titles and abstracts. If the information in the titles and abstracts was insufficient, we read the full text to determine whether the literature was included. Disagreements between the researchers when screening the literature were resolved via discussion. If necessary, a third researcher was consulted to reach a consensus.

### 2.3. Data Extraction

The following data were extracted from the included studies: (1) basic information—first author, publication year and month, country, and study design; (2) characteristics of participants—sample sizes and age groups; (3) information of the SARS-CoV-2 vaccines—name and the number of doses; (4) outcomes for the effectiveness of SARS-CoV-2 vaccines—the number of SARS-CoV-2 infection and laboratory-confirmed COVID-19; and (5) outcomes for the safety of SARS-CoV-2 vaccines—the number and kinds of adverse events after the first and second vaccination. The whole process of information extraction was completed independently by two investigators.

### 2.4. Risk of Bias Assessment

Two researchers independently assessed the methodological quality of the included studies by using the following tools: the Cochrane Risk of Bias tool for RCTs [18], the Newcastle Ottawa scale (NOS) for cohort studies [19], and the checklist recommended by Agency for Healthcare Research and Quality (AHRQ) for cross-sectional studies [20]. Significantly, RCTs were classified as having low (low risk of bias for all key domains), unclear (low or unclear risk of bias for all key domains), and high (high risk of bias for one or more key domains) risk of bias; cohort studies were classified as having low (7–9 stars), moderate (5–6 stars), and high risk of bias (0–4 stars); cross-sectional studies were classified as having low (8–11 scores), moderate (4–7 scores), and high risk of bias (0–3 scores).

### 2.5. Data Synthesis and Statistical Analysis

We calculated the vaccine efficacy against SARS-CoV-2 infection and COVID-19 to assess the effectiveness of SARS-CoV-2 vaccines, and incidence rate of adverse events to assess safety of SARS-CoV-2 vaccines at the level of “cohort”. Participants with different ages, follow-up time, or who received different dosage of vaccines were regard as different cohorts independently. Vaccine efficacy was defined as 100* (1-RR), where RR (risk ratio) is the ratio of rate of SARS-CoV-2 infection (or prevalence of COVID-19) in the vaccinated group to the corresponding rate in the unvaccinated group. Data of the first dose, second, and third dose were pooled in meta-analysis separately. We calculated I^2^ statistics and conducted χ^2^ test to assess the heterogeneity between studies. We chose fixed-effect models to pool studies if I^2^ ≤ 50% and *p*-value for χ^2^ test ≥ 0.05, which represented low-to-moderate heterogeneity. Otherwise, we chose random-effects models if I^2^ > 50% and *p* value for χ^2^ test < 0.05, which represented substantial heterogeneity. Stata (version 16.0) was used to analyze the data.

## 3. Results

### 3.1. Characteristics of Included Studies

**Figure 1:**
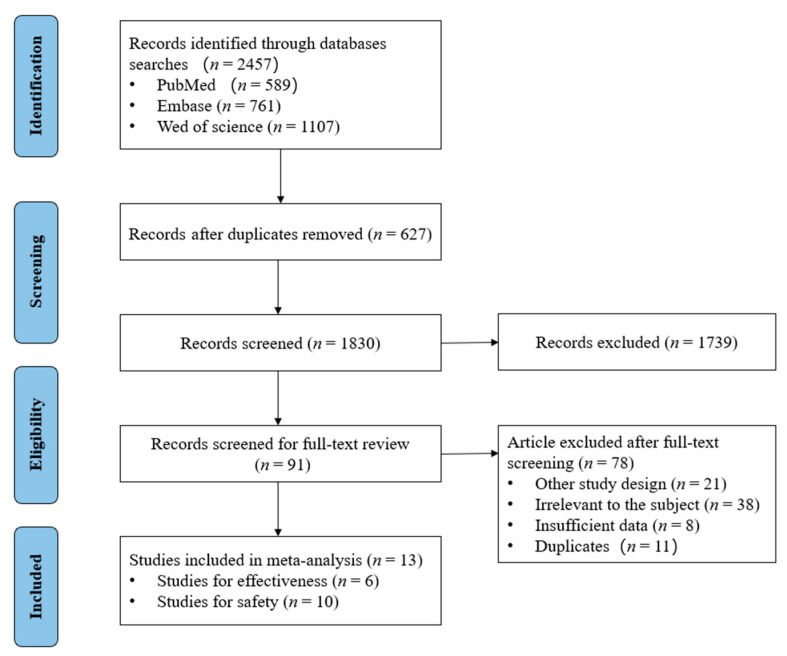
Flowchart of studies selection.

A total of 2457 citations were searched for PubMed, Embase, and Web of Science (Figure 1). Then 627 citations were excluded for duplicates. After the review by titles and abstracts, 91 citations were retained for full-text review. Finally, 13 eligible articles were reserved, including seven RCTs, three cohort studies, and three cross-sectional studies. Six articles were for the effectiveness (Supplementary Table S2) and ten articles were for safety of SARS-CoV-2 vaccines (Supplementary Table S3). Among studies giving an analysis of effectiveness, children and adolescents in four of those studies were from the USA (207,859 participants) and two studies were from multiple countries (3003 participants). Among studies for the analysis of safety, children and adolescents in four of those studies were form the USA (177,622 participants), four studies were from China (2796 participants), one study was from multiple countries (2260 participants), and one study was from Saudi Arabia (965 participants). Participants received the BNT162b2 mRNA COVID-19 vaccine in nine studies, (five studies for Americans, two studies for multinational participants, one study for Chinese, and one study for Saudi Arabian); the mRNA-1273 vaccine in one study for Americans; and the CoronaVac, BBIBP-CorV, and Ad5-vectored COVID-19 vaccines in three studies for Chinese separately. All of the studies had low risk of bias, except for one study with moderate risk of bias (Supplementary Table S4), indicating that their quality was high.

### 3.2. Vaccine Effectiveness

All results of vaccine effectiveness are summarized in Table 1. For the first dose of SARS-CoV-2 vaccines, the pooled vaccine efficacy against SARS-CoV-2 infection was 88.5% (95% CI:15.7–98.4%), corresponding to RR = 0.115 (95% CI:0.016–0.843, *p =* 0.033) with significance. The pooled vaccine efficacy against COVID-19 was 84.3% (95% CI:66.6–92.6%), corresponding to RR = 0.157 (95% CI:0.074–0.334, *p <* 0.001) with significance.

For the second dose of SARS-CoV-2 vaccines, the pooled vaccine efficacy against SARS-CoV-2 infection was 91.6% (95% CI:37.8–99.5%), corresponding to RR = 0.084 (95% CI:0.005–1.378, *p =* 0.083) without significance. The pooled vaccine efficacy against COVID-19 was 92.7% (95% CI:82.2–97.0%), corresponding to RR = 0.073 (95% CI:0.030–0.178, *p <* 0.001) with significance.

### 3.3. Vaccine Safety

The incidence rates of the kinds of adverse events after the first dose of SARS-CoV-2 vaccines were quite different, as shown in Table 2. Injection-site pain (33.4%), fatigue (27.8%), headache (18.8%), anorexia (16.7%), axillary swelling (14.2%), myalgia/muscle pain (12.7%), and chills (10.2%) were the most common adverse events. However, among them, only two cohorts reported axillary swelling and three cohorts reported anorexia. In addition, the incidence rate of any local adverse events (30.7%), any systemic adverse events (26.6%), and any adverse events (20.4%) was also high.

For the second dose of SARS-CoV-2 vaccines, fatigue (41.0%), injection-site pain (39.2%), headache (29.9%), chills (25.4%), myalgia/muscle pain (17.2%), axillary swelling (11.0%), fever (10.6%), and arthralgia/joint pain (10.3%) were common adverse events. They occurred more often than that after the first dose, except for axillary swelling. The incidence rates of any local adverse events (29.1%), any systemic adverse events (20.8%), and any adverse events (14.8%) had a decrease compared with which after the first dose.

For the third dose of SARS-CoV-2 vaccines, only the occurrence of cough is more frequent compared with it after the second dose. The incidence rates of other adverse events were lower than those after the first and second dose.

In addition, we conducted an analysis of safety of BNT162b2 COVID-19 vaccine (Supplementary Table S5). The safety of other vaccines was not analyzed separately, due to the lack of data. For the first dose, injection-site pain (60.5%), fatigue (34.3%), headache (26.8%), and myalgia/ muscle pain (16.1%) were common adverse events. Moreover, the incidence rates of them were higher than overall rates after the first dose of SARS-CoV-2 vaccines, except for chills.

For the second dose of the BNT162b2 COVID-19 vaccine, all kinds of adverse events occurred more often compared with that after the first dose of the BNT162b2 COVID-19 vaccine. Injection-site pain (62.7%), fatigue (49.5%), headache (42.4%), chills (23.5%), myalgia/muscle pain (22.1%), fever (19.7%), and nausea (13.8%) were common adverse events.

## 4. Discussion

To our knowledge, this is the first meta-analysis to evaluate the effectiveness and safety of SARS-CoV-2 vaccines among children and adolescents. After screening, 13 studies of children and adolescents aged 3–18 were included in this article, six articles for effectiveness comprehensive analysis and 10 articles for safety comprehensive analysis. In terms of effectiveness, our study showed that the effectiveness of the first and second doses of vaccines against SARS-CoV-2 infection was 88.5% and 91.6%, respectively, and the effectiveness against COVID-19 were 84.3% and 92.7%. The pooled vaccine efficacy against COVID-19 was significant after the first and second injection. In terms of safety, our study showed that, after the first dose of vaccination, the overall adverse-reaction rate was 20.4%, the local adverse-reaction rate was 30.7%, and the systemic adverse-reaction rate was 26.6%. Common adverse events included injection-site pain, fatigue, and headache. The incidence rate of common adverse events after the second dose of vaccination was generally higher than that of the first dose, which was 30–40%. Moreover, a stratified analysis of the safety data of the BNT162b2 vaccine showed that adverse events after the second dose were more frequent than those after the first dose.

In terms of the effectiveness against SARS-CoV-2 infection in children and adolescents, the pooled vaccine efficacy of three studies was not statistically significant compared with the control group for the second dos. Tartof et al. conducted a retrospective cohort study on the BNT162b2 vaccine [13], Ali et al. conducted a RCT on the effectiveness and safety of the mRNA-1273 vaccine in children and adolescents aged 12–17 [7], and Lutrick et al. conducted an interim estimate of a prospective cohort on the effectiveness of BNT162b2 vaccine among adolescents aged 12–17 years [21]. The three studies themselves showed that the vaccines had statistical significance compared with the control group in preventing SARS-CoV-2 infection in children and adolescents. The reason why the pooled vaccine efficacy was not significant may be that the research types of the three studies were different and the vaccines were different; that is, there were too few studies and too high heterogeneity among the three included studies; in terms of the effectiveness against COVID-19, the pooled vaccine (BNT162b2 and mRNA-1273 vaccines) efficacy of the first and second doses of vaccines in four studies was 84.3% and 92.7%, respectively, with significance compared with the control group [7,8,10,22].According to research, the effectiveness of the first and second doses of the BNT162b2 vaccine in effectiveness against COVID-19 in the real world was 53% and 95% [23], and the effectiveness against COVID-19 of the first and second doses of the mRNA-1273 vaccine were 95.2% and 93.2% [24,25]. It indicated that the BNT162b2 and mRNA-1273 vaccines had great effectiveness against COVID-19 in children and adolescents, close to the overall effectiveness of the current real-world SARS-CoV-2 vaccines. South Africa reported the Omicron (B1.1.529) variant to WHO in 24 November 2021 for the first time [26]. Effectiveness and safety of vaccines in this meta-analysis did not target Omicron because of the publication time of studies included.

The safety of vaccines in children and adolescents is the focus of general attention, and it is also an important factor affecting the vaccination rate [27]. A meta-analysis of the safety of SARS-CoV-2 vaccines in randomized controlled trials showed that the incidence rates of adverse events of inactivated vaccine, mRNA vaccine, and viral vector vaccine were 23.0%, 48.0%, and 76.0%—significantly higher compared with control group [28]. Based on VAERS, the United States conducted a safety review on American adolescents aged 12–17 who received BNT162b2 vaccine from 14 December 2020 to 16 July 2021, and the statistical results showed that 8.9 million adolescents who received the BNT162b2 vaccine would probably have 9246 adverse events, of which 90.7% were non-serious adverse events and 9.3% were serious adverse events, and systemic reactions were more common after the second dose [14]. Our article showed that the incidence rate of local and systemic adverse events of SARS-CoV-2 vaccines among children and adolescents were about 30%. The common ones were injection-site pain, headache, fatigue, chills, and so on. It showed that the safety of SARS-CoV-2 vaccines among children and adolescents was good, and the incidence rate of adverse events was at a normal level. For the safety of the vaccines, another focus should be on serious adverse events. A meta-analysis of the safety of SARS-CoV-2 vaccines in the real world showed that the overall pooled incidence of serious adverse events was 0.4 (0.2–0.5)/10,000 [29]. Limited by the number of studies, our article did not analyze the overall pooled incidence of serious adverse events. However, in terms of the included studies in our article, grade 3 adverse events, such as fever, injection-site pain, headache, fatigue, and chills, were the few serious adverse events that occurred, generally no more than 10% [7,8,9,10,11,12], and there were only cases of grade 4 adverse events [7]. Besides, among these included studies, a case of grade 3 allergic purpura occurred in the study of Xia et al. [11] and a case of gastrointestinal disorder occurred in the study of Zhu et al. [12] were both considered to be serious adverse events of great concern related to SARS-CoV-2 vaccines. There were no reports of myocarditis and pericarditis in our study, but this did not mean that myocarditis or pericarditis was not worthy of attention. According to the survey, 350 cases of myocarditis occurred among 8.9 million adolescents who have been vaccinated with BNT162b2 vaccine in the United States [14]; there were 97 males and 16 females with pericarditis, and myocarditis occurred in every million 12–17 year-old adolescents vaccinated with BNT162b2 vaccine in Denmark [30]. It reminded us that it was still necessary to monitor and follow up serious adverse events in children and adolescents vaccinated with SARS-CoV-2 vaccines, especially adverse events, such as pericarditis, myocarditis, and allergic purpura.

With the popularity of SARS-CoV-2 vaccines among adults, the proportion of children and adolescents infected with SARS-CoV-2 has gradually increased. Monitoring data in the United States showed that, between 1 March 2020 and 14 August 2021, the cumulative incidence rate of COVID-19-related hospitalizations among children and adolescents was 49.7 per 100,000 persons. In the week ending on 14 August 2021, the weekly hospitalization rate (1.4) related to COVID-19 among children and adolescents was almost five times that of the week ending 26 June 2021 (0.3), and the risk of hospitalization related to COVID-19 for non-vaccinated adolescents was 10.1 times higher than that of fully vaccinated adolescents [31]. In order to effectively control the spread of SARS-CoV-2, more and more countries began to approve the use of SARS-CoV-2 vaccines in children and adolescents. Most Western countries have approved the emergency use of mRNA vaccines (BNT162b2 and mRNA-1273 vaccines) in children and adolescents in 2021 [17], during which the BNT162b2 vaccine was the most widely used vaccine approved for children and adolescents [8,10,13,21,22,32,33,34,35]. China approved the emergency use of the SARS-CoV-2 inactivated vaccine for children and adolescents aged 3–17 years in July 2021, and has completed over 60 million doses of SARS-CoV-2 vaccines for adolescents aged 12–17 years [36]. However, there were many surveys completed on the willingness of children and adolescents to be vaccinated with SARS-CoV-2 vaccines in the United States, Germany, South Korea, the United Kingdom, and other countries, showing that only 40–60% of children and adolescents were willing to get vaccinated against COVID-19, and most of the reasons for hesitation in vaccination were concerns about the effectiveness and safety of SARS-CoV-2 vaccines [37,38,39,40]. To sum up, many countries have approved the emergency use of SARS-CoV-2 vaccines in children and adolescents, but the available data related to the application of SARS-CoV-2 vaccines in children and adolescents are limited, especially the effectiveness of SARS-CoV-2 vaccines. Moreover, there is not much evidence-based medicine evidence. Thus, more basic research and clinical trials are still needed to explore the effectiveness, immunogenicity, and safety of SARS-CoV-2 vaccines among children and adolescents. In addition, many studies have shown that children and adolescents are not willing to be vaccinated with SARS-CoV-2 vaccines, and there are many influencing factors, among which worrying about the effectiveness and safety of the vaccine is an essential factor for hesitating to vaccinate. Therefore, it is recommended to conduct a systematic review of the factors that affect the COVID-19 vaccination, the willingness of COVID-19 vaccination, and the methods to promote COVID-19 vaccination of children and adolescents in order to promote the use of SARS-CoV-2 vaccines in children and adolescents.

There are also some limitations in our article. Firstly, the number of studies used for our comprehensive analysis of vaccine effectiveness is small, and the heterogeneity of included studies is large, making the comprehensive analysis results controversial and, thus, in need of further verification. Secondly, most of the safety data are adverse events within 7 days, which may lead to an underestimation of the incidence rate of adverse events. Thirdly, due to the different measurement methods and indicators of the immunogenicity of SARS-CoV-2 vaccines in various included studies, the comprehensive analysis of the immunogenicity of SARS-CoV-2 vaccines in children and adolescents has not been carried out. Finally, there was great heterogeneity in included studies, and because of the number of studies, we did not conduct the subgroup analysis based on the type of vaccines, the age of participants, and so on.

## 5. Conclusions

As far as current research is concerned, the SARS-CoV-2 vaccines have good effectiveness and safety in children and adolescents. SARS-CoV-2 vaccines can effectively prevent SARS-CoV-2 infection among children and adolescents, and most of the observed adverse events are mild. However, there are currently few studies on the effectiveness and safety of SARS-CoV-2 vaccines in children and adolescents, and the available data are limited, especially the effectiveness of SARS-CoV-2 vaccines. More basic research and clinical trials are still needed to explore its effectiveness, immunogenicity, and safety.

## Figures and Tables

**Table 1:** Vaccine effectiveness of SARS-CoV-2 vaccines.

	No. of Cohorts	RR (95% CI)	Vaccine Effectiveness (%) (95% CI)	*p*-Value of Meta-Analysis	I^2^ (%)
First dose					
*Efficacy against SARS-CoV-2 infection*	2	0.115 (0.016–0.843)	88.5 (15.7–98.4)	0.033	97.6
*Efficacy against COVID-19*	3	0.157 (0.074–0.334)	84.3 (66.6–92.6)	<0.001	0.0
Second dose					
*Efficacy against SARS-CoV-2 infection*	3	0.084 (0.005–1.378)	91.6 (37.8–99.5)	0.083	98.7
*Efficacy against COVID-19*	5	0.073 (0.030–0.178)	92.7 (82.2–97.0)	<0.001	0.0

**Table 2:** Incidence rates of adverse events after each dose of SARS-CoV-2 vaccines.

Adverse Events	First-Dose			Second-Dose			Third-Dose		
	No. of Cohorts	Incidence Rate of Adverse Events (%) (95% CI)	I^2^ (%)	No. of Cohorts	Incidence Rate of Adverse Events (%) (95% CI)	I^2^ (%)	No. of Cohorts	Incidence Rate of Adverse Events (%) (95% CI)	I^2^ (%)
Nausea	7	5.3 (3.8–7.4)	99.4	7	6.9 (4.3–10.7)	99.7			
Vomiting	14	2.0 (1.4–2.7)	97.1	11	3.2 (2.4–4.4)	96.9			
Diarrhea	13	3.8 (3.0–4.8)	96.3	9	4.1 (3.0–5.7)	98.1			
Anorexia	3	16.7 (0.4–8.9)	81.5	3	1.2 (0.5–2.6)	0.0			
Headache	18	18.8 (14.8–23.4)	99.6	15	29.9 (22.2–38.8)	99.8			
Injection-site pain	22	33.4 (29.3–37.8)	99.4	19	39.2 (34.6–44.0)	99.1	4	1.6 (0.7–3.8)	0.0
Myalgia/muscle pain	11	12.7 (9.0–17.7)	99.8	12	17.2 (11.3–25.5)	99.9			
Arthralgia/joint pain	11	6.3 (4.3–9.1)	99.4	7	10.3 (6.3–16.5)	99.8			
Fever	22	7.6 (6.7–8.6)	94.8	19	10.6 (8.0–13.7)	99.5	7	1.7 (0.9–3.1)	0.0
Cough	10	3.5 (2.4–4.9)	25.4	10	1.9 (1.2–2.9)	0.0	5	2.2 (1.1–4.2)	0.0
Chills	10	10.2 (7.4–13.9)	99.5	11	25.4 (18.1–34.5)	99.8			
Fatigue	15	27.8 (23.1-33.1)	99.6	13	41.0 (32.8–49.9)	99.8			
Rash	3	1.2 (1.1–1.3)	0.0	3	1.1 (1.0–1.2)	69.1			
Swelling	14	5.8 (4.6–7.6)	98.8	14	6.7 (5.0–8.8)	98.9			
Axillary swelling	2	14.2 (-3.6–32.0)	99.7	2	11.0 (-8.5–30.5)	99.8			
Itching	5	4.9 (3.8–6.0)	98.4	5	4.8 (3.7–6.3)	98.3	2	1.2 (0.3–4.7)	0.0
Pruritus	3	1.0 (0.4–2.4)	0.0	2	0.5 (0.1–1.9)	0.0			
Redness	11	4.9 (4.0–6.1)	96.6	15	6.4 (5.1–7.9)	95.5	4	2.7 (1.4–5.3)	0.0
Erythema	2	7.0 (-5.6–19.6)	99.7	3	1.5 (0.0–36.2)	98.6			
Mucocutaneous eruption	2	0.7 (0.2–2.2)	0.0						
Induration	3	1.3 (0.6–3.1)	0.0						
Abdominal pain	3	4.6 (4.1–5.2)	96.8	3	7.3 (6.1–8.5)	98.4			
Dyspnea				2	1.1 (0.3–4.2)	0.0			
Any local adverse events	14	30.7 (25.8–36.1)	99.6	14	29.1 (15.0–28.1)	99.4	5	4.3 (2.6–6.9)	0.0
Any systemic adverse events	14	26.6 (21.7–32.2)	99.7	14	20.8 (15.0–28.1)	99.8	8	4.0 (2.6–6.1)	39.7
Any adverse events	16	20.4 (8.6–41.2)	99.2	16	14.8 (5.0–36.5)	99.2	9	5.8 (4.3–8.0)	42.8

## Data Availability

Data can be obtained by contacting the corresponding author.

## Supplementary Materials

The following supporting information can be downloaded at: https://www.mdpi.com/article/10.3390/vaccines10030421/s1, Table S1: Search strategy. Table S2: Articles for analysis of effectiveness. Table S3: Articles for analysis of safety. Table S4: Results of quality assessment. Table S5: Incidence rates of adverse events after each dose of BNT162b2.
