# Supplementary material for: Effectiveness and Safety of SARS-CoV-2 Vaccines among Children and Adolescents: A Systematic Review and Meta-Analysis
Source: Vaccines (Basel). 2022 Mar 10;10(3):421. doi: 10.3390/vaccines10030421 (PMC8953823; doi:10.3390/vaccines10030421)
Supplement: Supplementary file 1 [file vaccines-10-00421-s001.zip › vaccines-1621130-supplementary.pdf]

Table S1. Search strategy

Databases	Detailed search strategy
Pubmed	((((Vaccines[MeSH Terms]) OR (Vaccination[MeSH Terms]) OR (Vaccine[Title/Abstract]) OR (Vaccination[Title/Abstract]) OR (Active Immunization[Title/Abstract])) AND ((COVID-19[MeSH Terms]) OR (SARS-CoV-2[MeSH Terms]) OR (COVID-19[Title/Abstract]) OR (SARS-CoV-2[Title/Abstract]) OR (2019 novel coronavirus[Title/Abstract]) OR (2019 novel coronaviruses[Title/Abstract]) OR (COVID19[Title/Abstract]) OR (2019-nCoV[Title/Abstract]) OR (coronavirus disease 2019[Title/Abstract]) OR (coronavirus disease 2019 virus[Title/Abstract]) OR (SARS Coronavirus 2[Title/Abstract]) OR (Coronavirus Disease-19[Title/Abstract]) OR (SARS CoV 2 Virus[Title/Abstract]))) AND ((Child[MeSH Terms]) OR (Infant[MeSH Terms]) OR (Adolescent[MeSH Terms]) OR (Child[Title/Abstract]) OR (Children[Title/Abstract]) OR (Childhood[Title/Abstract]) OR (Infant[Title/Abstract]) OR (Adolescent[Title/Abstract]) OR (Adolescence[Title/Abstract]) OR (Teenager[Title/Abstract]) OR (Youth[Title/Abstract]))) AND ((Safety[MeSH Terms]) OR (Drug-Related Side Effects and Adverse Reactions[MeSH Terms]) OR (safety[Title/Abstract]) OR (effectiveness[Title/Abstract]) OR (safe[Title/Abstract]) OR (safeties[Title/Abstract]) OR (efficacy[Title/Abstract]) OR (adverse

	effect[Title/Abstract]) OR (adverse event[Title/Abstract]) OR (side effect[Title/Abstract]) OR (tolerability[Title/Abstract]))
Embase	((('vaccine'/exp) OR ('vaccination'/exp) OR ((’vaccine’ OR ‘vaccination’ OR ‘active immunization’):ab,ti)) AND (('coronavirus disease 2019'/exp) OR ('Severe acute respiratory syndrome coronavirus 2'/exp) OR ((’COVID-19’ OR ‘SARS-CoV-2’ OR ‘2019 novel coronavirus’ OR ‘2019 novel coronaviruses’ OR ‘COVID19’ OR ‘2019-nCoV’ OR ‘coronavirus disease 2019’ OR ‘SARS Coronavirus 2’ OR ‘Coronavirus Disease-19’ OR ‘SARS CoV 2 Virus’):ab,ti))) AND (('child'/exp) OR ('adolescent'/exp) OR ('child'/exp) OR ('juvenile'/exp) OR ((’child’ OR ‘infant’ OR ‘adolescent’ OR ‘juvenile’ OR ‘children’ OR ‘childhood’ OR ‘adolescence’ OR ‘adolescent’ OR ‘teenager’ OR ‘youth’):ab,ti)) AND (('safety'/exp) OR ('adverse event'/exp) OR ('side effect'/exp) OR ((’safety’ OR ‘Drug-Related Side Effects and Adverse Reactions’ OR ‘effectiveness’ OR ‘safe’ OR ‘safeties’ OR ‘effectiveness’ OR ‘efficacy’ OR ‘adverse effect’ OR ‘adverse event’ OR ‘side effect’ OR ‘tolerability’):ab,ti))
Wed of science	TS=((vaccine OR vaccination OR "active immunization" OR "active immunizations") AND ("COVID-19" OR "SARS-CoV-2" OR "2019 novel coronavirus" OR "2019 novel coronavirus" OR COVID19 OR "2019-nCoV" OR "coronavirus disease 2019" OR “SARS Coronavirus 2” OR “Coronavirus Disease-19” OR “SARS CoV 2 Virus” OR “Severe acute respiratory syndrome coronavirus 2”) AND (child OR infant OR juvenile OR

	children OR childhood OR adolescence OR adolescent OR teenager OR youth) AND (safety OR "Drug-Related Side Effects and Adverse Reactions" OR effectiveness OR safe OR safeties OR efficacy OR "adverse effect" OR "adverse event" OR "side effect" OR tolerability))

Table S2. Articles for analysis of effectiveness

First author	Publication time	Study design	Country of population	Kinds of SARS-CoV-2 vaccine	Vaccination status	Age of population (years old)	Vaccinated people with SARA-CoV-2 infection (n/N)	Unvaccinated people with SARA-CoV-2 infection (n/N)	Vaccinated people with COVID-19 (n/N)	Unvaccinated people with COVID-19 (n/N)
Sara Y Tartof	2021.10	cohort study	USA	BNT162b2	1 dose (<14days)	12-15	24/7164	8425/104918		
					2 dose (≥7days)	12-15	59/78843	8425/104918		
Kashif Ali	2021.8	RCT Phase 2-3	USA	mRNA-1273 vaccine	1 dose (>14days)	12-17	27/2163	42/1073	2/2163	13/1073
					2 dose (1>4days)	12-17	22/2139	23/1024	1/2139	7/1042
Robert W. Frenck, Jr.	2021.5	RCT Phase 3	multinational	BNT162b2	1 dose (<21days)	12-15			3/1131	12/1129

					2 dose (<7days)	12-15			0/1131	5/1129
					2 dose (>7days and <4months)	12-15			0/1131	18/1129
E.B. Walter	2021.11	RCT Phase 2-3	USA	BNT162b2	2 dose (>7days)	5-11			3/1045	16/736
Karen Lutrick	2021.12	cohort study	USA	BNT162b2	2 dose (≥14days)	12-17	5/190	16/66		
S.J. Thomas	2021.9	RCT Phase 2-3	multinational	BNT162b2	1 dose (≥7days)	16-17			3/373	19/370
					2 dose (≥7days)	16-17			0/342	10/331

Table S3. Articles for analysis of safety

First author	Publication time	Study design	Country of population	Kinds of SARS-CoV-2 vaccine	Vaccination status	Dosage	Age of population (years old)	Sample size (N)	Adverse events																									
									Nausea	Vomiting	Diarrhoea	Anorexia	Headache	Injection site pain	Myalgia/ Muscle pain	Arthralgia/ Joint pain	Fever	Cough	Dyspnea	Chills	Fatigue	Rash	Swelling	Axillary Swelling	Itching	Pruritus	Redness	Erythema	Mucocutaneous eruption	Induration	Abdominal pain	Any local adverse events	Any systemic adverse events	Any adverse events
Anne M. Hause	2021.8	cross-sectional study	USA	BNT162b2	1 dose (≤7days)		12-15	62709	4703	627	1944		15803	38378	13420	3951	5832			4264	17182	753	4703		3637		2571				2571	40071	30665	
					1 dose (≤7days)		16-17	66350	6768	730	2787		19772	39943	16853	5242	6569			5507	22625	796	5109		3782		2256				3118	41601	36957	
					2 dose (≤7days)		12-15	38817	5745	1009	1281		16963	23251	12189	4813	11606			8190	17312	467	3455		2135		2057				2717	24222	24610	
					2 dose (≤7days)		16-17	41040	8126	944	2011		20766	25445	16703	7469	12722			10752	21464	451	4063		2586		2011				3488	26430	28687	
Kashif Ali	2021.8	RCT Phase 2-3	USA	mRNA-1273 vaccine	1 dose (≤7days)		12-17	2482					1106 (N=2480)	2310	668 (N=2480)	371 (2480)	63 (2480)			456 (N=2480)	1188 (N=2481)		403	578				334				2339	1701	2381
					1 dose (>7days)		12-17	2482						20									18	127				15				160		

					2 dose (≤7days)		12-17	2478					1739	2290	1154 (N=2477)	716 (N=2477)	302 (N=2477)			1066 (N=2477)	1679		509	519 (N=2477)				484				2314	2134	2405
					2 dose (>7days)		12-17	2478						11									6	27				6				40		
Robert W. Frenck, Jr.	2021.5	RCT Phase 3	multinational	BNT162b2	1 dose (≤7days)		12-15	1131		34	90		622	973	271	113	113			317	679		79				68							
					2 dose (≤7days)		12-15	1131		34	68		735	893	362	181	226			475	746		57				57							
Bihua Han	2021.1	RCT Phase 1-2	China	CoronaVac	1 dose (≤28days)	low dose	3-17	219	2	1	1	2	4	19	0		6	3			1		1			3			1	0				33
					1 dose (≤28days)	high dose	3-17	217	2	1	2	2	2	21	0		10	6			1		3			1			2	2				46
					2 dose (≤28days)	low dose	3-17	214	2	2	1	2	3	25	1		3	3			1		2			1		0	1					35
					2 dose (≤28days)	high dose	3-17	211	1	0	0	2	2	20	0		2	2			0		4			1		1	0					26
E.B.	2021.11	RCT	USA	BNT16	1 dose	1 dose with low	5-11	16		0	1		4	14	2	1	1			0	8		3				2							

Walter	Phase 2-3	2b2	(≤7days) in the phase 1 study	dose and 2 dose with low dose													
			1 dose (≤7days) in the phase 1 study	1 dose with medium dose and 2 dose with medium dose	5-11	16	1	1	5	15	4	1	1	4	11	1	0
			1 dose (≤7days) in the phase 1 study	1 dose with high dose and 2 dose with high dose	5-11	4	0	0	3	4	4	1	0	2	4	2	4
			1 dose (≤7days) in the phase 1 study	1 dose with high dose and 2 dose with low dose	5-11	12	1	0	4	10	0	1	4	2	6	1	2
			2 dose (≤7days) in the phase 1 study	1 dose with low dose and 2 dose with low dose	5-11	16	0	1	8	14	0	0	2	5	11	5	6
			2 dose (≤7days) in the phase 1 study	1 dose with medium dose and 2 dose with medium dose	5-11	16	0	0	9	12	3	0	3	7	10	3	3
			2 dose (≤7days) in the	1 dose with high dose and 2 dose	5-11	4	1	2	3	4	2	1	4	3	4	2	3

					phase 1 study	with high dose																		
					2 dose (≤7days) in the phase 1 study	1 dose with high dose and 2 dose with low dose	5-11	12	1	0	4	11	1	0	0		4	9	0	2				
					1 dose (≤7days) in the phase 2-3 study	low dose	5-11	1511	26	85	359	915	133	60	29		77	501	125	176				
					2 dose (≤7days) in the phase 2-3 study	low dose	5-11	1501	17	88	379	849	155	73	79		131	527	164	211				
ShengLi Xia	2021.9	RCT Phase1-2	China	BBIBP-CorV	1 dose (≤7days)	low dose	3-5	83	0	0	1	0			4	5		0	0	0		0	10	10 (≤30days after 1 dose)
					1 dose (≤7days)	medium dose	3-5	84	1	1	0	3			7	5		0	0	0		3	14	18 (≤30days after 1 dose)
					1 dose (≤7days)	high dose	3-5	84	0	0	2	3			11	2		1	0	1		4	16	20 (≤30days after 1 dose)
					1 dose (≤7days)	low dose	6-12	84	0	1		2			1	1				0	0	2	3	5 (≤30days after 1 dose)

1 dose<br>(≤7days)	medium dose	6-12	84		0	0		5		1	1				2	1	8	3	11 (≤30days<br>after 1 dose)
1 dose<br>(≤7days)	high dose	6-12	84		2	0		4		6	2				0	0	4	10	15 (≤30days<br>after 1 dose)
1 dose<br>(≤7days)	low dose	13-17	84	2	2	0	1	4	3	6	0	3	0	0	0		4	18	24 (≤30days<br>after 1 dose)
1 dose<br>(≤7days)	medium dose	13-17	84	0	0	4	1	8	0	6	1	0	0	1	0		9	12	21 (≤30days<br>after 1 dose)
1 dose<br>(≤7days)	high dose	13-17	84	0	0	0	0	3	0	1	0	0	0	0	1		4	1	5 (≤30days<br>after 1 dose)
2 dose<br>(≤7days)	low dose	3-5	83					0		0	1	0			1		1	1	2 (≤30days<br>after 2 dose)
2 dose<br>(≤7days)	medium dose	3-5	83					0		6	0	0			1		1	6	7 (≤30days<br>after 2 dose)
2 dose<br>(≤7days)	high dose	3-5	83					2		2	2	0			0		2	4	6 (≤30days<br>after 2 dose)
2 dose<br>(≤7days)	low dose	6-12	84				0	2		1	1		0	0	0		2	3	5 (≤30days<br>after 2 dose)
2 dose<br>(≤7days)	medium dose	6-12	83				0	4		0	2		1	1	1		7	2	9 (≤30days<br>after 2 dose)
2 dose	high dose	6-12	83				0	4		3	0		0	0	2		6	3	9 (≤30days

	(≤7days)																			after 2 dose)
	2 dose (≤7days)	low dose	13-17	84	0	0		1	0	1	3	1	1	1			1	1	8	9 (≤30days after 2 dose)
	2 dose (≤7days)	medium dose	13-17	83	0	0		0	4	0	2	1	0	0			0	4	3	7 (≤30days after 2 dose)
	2 dose (≤7days)	high dose	13-17	84	2	2		0	0	0	4	1	0	0			2	0	9	11 (≤30days after 2 dose)
	3 dose (≤7days)	low dose	3-5	82		0	0	0	0		1	2		0				0	3	3 (≤30days after 3 dose)
	3 dose (≤7days)	medium dose	3-5	83		0	1	0	0		1	0		0				0	2	2 (≤30days after 3 dose)
	3 dose (≤7days)	high dose	3-5	83		1	0	1	2		2	3		0				2	8	10 (≤30days after 3 dose)
	3 dose (≤7days)	low dose	6-12	83					1		0	0			1	1	1	4	0	4 (≤30days after 3 dose)
	3 dose (≤7days)	medium dose	6-12	84					1		0	1			0	0	3	4	1	5 (≤30days after 3 dose)
	3 dose (≤7days)	high dose	6-12	83					0		1	0			0	0	1	1	1	2 (≤30days after 3 dose)
	3 dose (≤7days)	low dose	13-17	84					1		2	0				1	3	5	2	7 (≤30days after 3 dose)

					3 dose (≤7days)	medium dose	13-17	83						0			1	1					0		0		0	2	2 (≤30days after 3 dose)
					3 dose (≤7days)	high dose	13-17	84						0			1	1					0		0		0	2	2 (≤30days after 3 dose)
Fengcai Zhu	2021.9	RCT Phase 2b	China	Ad5-vectored COVID-19 vaccine	1 dose (≤14days)		6-17	100	4	2	2	7	14	34	4	1	28	6	0	9	4	2	1	2	2	36	37	55
					1 dose (>14days)		6-17	100																				4
					2 dose (≤14days)		6-17	100	3	2	1	2	8	14	1	1	13	4	1	6	4	3	0	0	1	19	24	33
					2 dose (>14days)		6-17	100																				3
Edward Wai Wa Chan	2021.12	cohort study	China	BNT162b2	1 dose (≤7days)		12-17	1016																				50
					2 dose (≤7days)		12-17	1016																				72

Anne M. Hause	2021.12	cross-sectional study	USA	BNT162b2	1 dose (≤7days)	5-11	42504	2125	987	1105	5908	22400	3018	893	3358	1658	8543	510	1658	1615	1573	2168	23290	14734	
					2 dose (≤7days)	5-11	29899	2063	807	658	5920	16684	3050	867	4006	2033	7744	299	1465	1106	1316	1914	17192	12229	
Edrous Alamer	2021.11	cross-sectional study	Saudi Arabia	BNT162b2	1 dose	12-18	965		42		146	269			116	58	181								298
					2 dose	12-18	426		78		168	247			161	110	201								273

Table S4. Results of quality assessment

Quality assessment of cross-sectional study by AHRQ													
First author	Define the source of information (survey, record review)	List inclusion and exclusion criteria for exposed and unexposed subjects (cases and controls) or refer to previous publications	Indicate time period used for identifying patients	Indicate whether or not subjects were consecutive if not population-based	Indicate if evaluators of subjective components of study were masked to other aspects of the status of the participants	Describe any assessments undertaken for quality assurance purposes (e.g., test/retest of primary outcome measurements)	Explain any patient exclusions from analysis	Describe how confounding was assessed and/or controlled	If applicable, explain how missing data were handled in the analysis	Summarize patient response rates and completeness of data collection	Clarify what follow-up, if any, was expected and the percentage of patients for which incomplete data or follow-up was obtained	Total score	Risk of bias
Anne M. Hause	1	1	1	1	1	0	1	0	1	0	1	8	Low risk of bias
Edrous Alamer	1	1	1	0	1	1	1	0	1	1	1	9	Low risk of bias
Anne M. Hause	1	1	1	1	1	0	1	0	1	1	1	9	Low risk of bias
Allison L. Naleway	1	1	1	1	1	1	1	1	1	1	1	11	Low risk of bias

Quality assessment of cohort study by NOS										
First author	Selection				Comparability	Outcome			Total score	Risk of bias
	Representativeness of the exposed cohort	Selection of the non exposed cohort	Ascertainment of exposure	Demonstration that outcome of interest was not present at start of study	Comparability of cohorts on the basis of the design or analysis	Assessment of outcome	Was follow-up long enough for outcomes to occur	Adequacy of follow up of cohorts		
Sara Y Tartof	1	1	1	0	1	1	1	1	7	Low risk of bias

Edward Wai Wa Chan	0	1	1	1	2	0	1	0	6	Moderate risk of bias
Karen Lutrick	1	1	1	1	1	1	1	1	8	Low risk of bias

Quality assessment of RCTs by the Cochrane Risk of Bias tool

First author	Selection		Performance bias	Dectetion bias	Attrition bias	Reporting bias	Other bias	Risk of bias
	Random sequence generation	Allocation concealment	Blinding of outcome assessment	Blinding of outcome assessment	Incomplete outcome data	Selective reporting	Anything else, ideally prespecified	
Kashif Ali	Low	Low	Low	Low	Low	Low	Low	Low risk of bias
Robert W. Frenck, Jr.	Low	Low	Low	Low	Low	Low	Low	Low risk of bias
Bihua Han	Low	Low	Low	Low	Low	Low	Low	Low risk of bias
E.B. Walter	Low	Low	Low	Low	Low	Low	Low	Low risk of bias
ShengLi Xia	Low	Low	Low	Low	Low	Low	Low	Low risk of bias
S.J. Thomas	Low	Low	Low	Low	Low	Low	Low	Low risk of bias

Table S5. Incidence rates of adverse events after each dose of BNT162b2

Adverse events	The first dose			The second dose		
	NO. cohorts	Incidence rate of adverse events (%) (95% CI)	I^2 (%)	NO. cohorts	Incidence rate of adverse events (%) (95% CI)	I^2 (%)
Nausea	3	7.6 (4.7-10.4)	99.8	3	13.8 (6.2-21.4)	99.9
Vomiting	8	2.2 (1.4-3.1)	98.4	8	3.6 (2.5-4.9)	97.8
Diarrhoea	7	4.3 (3.4-5.50	98.1	7	4.5 (3.3-6.3)	98.5
Headache	8	26.8 (20.9-33.7)	99.8	10	42.4 (31.8-53.7)	99.9
Injection site pain	10	60.5 (56.0-64.8)	99.5	9	62.7 (59.6-65.7)	98.3
Myalgia/Muscle pain	7	16.1 (10.7-23.4)	99.9	8	22.1 (13.7-33.6)	99.9
Arthralgia/Joint pain	9	5.7 (3.8-8.5)	99.5	6	9.5 (5.4-16.0)	99.8
Fever	9	8.6 (7.6-9.7)	96.4	8	19.7 (14.2-26.5)	99.8
Chills	9	9.2 (6.6-12.4)	99.4	10	23.5 (16.4-32.4)	99.8
Fatigue	9	34.3 (28.4-40.7)	99.7	9	49.5 (39.9-59.2)	99.8
Rash	3	1.2 (1.1-1.3)	0.0			
Swelling	9	7.2 (5.7-9.3)	98.9	8	9.0 (6.9-11.6)	98.9
Itching	3	5.1 (3.8-6.4)	99.3	3	5.2 (3.6-6.7)	99.3
Redness	7	5.3 (4.3-6.5)	97.9	9	7.3 (5.8-9.2)	97.4
Abdominal pain	3	4.6 (4.1-5.2)	96.8	3	7.3 (6.1-8.5)	98.4
Any local adverse events	3	60.5 (55.4-65.6)	99.8	3	61.4 (57.6-65.3)	99.4
Any systemic adverse	3	46.4 (34.8-58.1)	100.0	3	58.1 (41.8-74.4)	100.0

events						
Any adverse events	2	17.9 (-7.6-43.3)	99.6	2	35.5 (-20.3-91.4)	99.8
